# Chitosan Modified by Kombucha-Derived Bacterial Cellulose: Rheological Behavior and Properties of Convened Biopolymer Films

**DOI:** 10.3390/polym14214572

**Published:** 2022-10-28

**Authors:** Hau Trung Nguyen, Alina Sionkowska, Katarzyna Lewandowska, Patrycja Brudzyńska, Marta Szulc, Nabanita Saha, Tomas Saha, Petr Saha

**Affiliations:** 1 Centre of Polymer Systems, University Institute, Tomas Bata University in Zlin, Tr. T. Bati 5678, 76001 Zlin, Czech Republic; 2 Institute of Biotechnology and Food Technology, Industrial University of Ho Chi Minh City, 12 Nguyen Van Bao, Ward 4, Go Vap District, Ho Chi Minh City 727000, Vietnam; 3 Department of Biomaterials and Cosmetics Chemistry, Faculty of Chemistry, Nicolaus Copernicus University in Toruń, Gagarina 7, 87-100 Toruń, Poland; 4 Footwear Research Centre, University Institute, Tomas Bata University in Zlin, Nad Ovcirnou IV 3685, 76001 Zlin, Czech Republic; 5 Faculty of Technology, Tomas Bata University in Zlin, Vavrečkova 275, 76001 Zlin, Czech Republic

**Keywords:** kombucha-derived bacterial cellulose, bacterial cellulose, chitosan, rheological properties, viscosity, biocomposite, film

## Abstract

This work investigates the rheological behavior and characteristics of solutions and convened biopolymer films from Chitosan (Chi) modified by kombucha-derived bacterial cellulose (KBC). The Arrhenius equation and the Ostwald de Waele model (power-law) revealed that the Chi/KBC solutions exhibited non-Newtonian behavior. Both temperature and KBC concentration strongly affected their solution viscosity. With the selection of a proper solvent for chitosan solubilization, it may be possible to improve the performances of chitosan films for specific applications. The elasticity of the prepared films containing KBC 10% *w*/*w* was preferable when compared to the controls. FTIR analysis has confirmed the presence of bacterial cellulose, chitosan acetate, and chitosan lactate as the corresponding components in the produced biopolymer films. The thermal behaviors of the Chi (lactic acid)/KBC samples showed slightly higher stability than Chi (acetic acid)/KBC. Generally, these results will be helpful in the preparation processes of the solutions and biopolymer films of Chi dissolved in acetic or lactic acid modified by KBC powder to fabricate food packaging, scaffolds, and bioprinting inks, or products related to injection or direct extrusion through a needle.

## 1. Introduction

In the context of the aggravation of environmental pollution and the energy crisis, the evolution of sustainable or renewable materials has been recorded as a global necessity to ensure a sustainable development society. Chitosan/bacterial cellulose (Chi/KBC) blends have also received particular research interest due to the unique structure and properties of individual components. Chitosan (Chi) is a linear polysaccharide consisting of randomly distributed β-(1→4)-linked D-glucosamine (deacetylated unit) and N-acetyl-D-glucosamine (acetylated unit) [1,2,3,4]. Chi has been verified as an important biomaterial with many commercial applications, such as tissue engineering, drug delivery, biomedical, food packaging, and chemical industries [5,6,7,8,9]. Chi is biodegradable, biocompatible, non-toxic, edible, antibacterial, and available in many physical forms [3,5,10,11]. Molecular weight, acetylation, and dissolved solvent play an important role in the viscosity, thermal stability, and mechanical properties of this polycationic biopolymer [2,3,4,5,6,10,11,12,13].

Bacterial cellulose (BC) is an emerging, extracellular natural polymer with polymerization degrees up to several million in β-1,4-linked glucopyranosyl chains [14,15]. BC is considered to be an unlimited raw material resource due to its distinguished properties, such as high purity, elasticity, durability, porosity, biodegradability, a high degree of crystallinity, good biocompatibility, non-toxicity, high thermal stability, and water holding capacity, as well as its three-dimensional fibrillar structure without lignin and hemicellulose [15,16,17,18,19,20,21,22,23]. Currently, BC is mainly biosynthesized through the control enzyme systems (cell-free BC synthesis) [24] or aerobic culture processes using different microorganism groups, including *Agrobacterium*, *Acetobacter*, *Gluconacetobacter*, *Komagataeibacter*, *Sarcina*, and *Pseudomonas* [25,26] in Hestrin and Schramm (HS) standard medium [27], as well as alternative inexpensive nutrient media prepared from rotten apple, pineapple, pomegranate, watermelon, tomato, orange fruits, potato peel wastes, sugarcane molasses, vinasse, distillery effluent, and the by-products of dairy foods [28,29,30,31,32,33,34,35,36]. In particular, BC has also been harvested from kombucha fermentation of a traditional beverage, which is simpler than all other cellulose production methods [37,38,39,40]. Kombucha fermentation is chemical-free and normally only requires a short-time fermentation of tea, sugar, and bio-wastes. According to structural analysis, these harvested kombucha-derived bacterial cellulose (KBC) possessed similar characteristics to the BC of HS standard medium or the alternative nutrient media [37,39,40]. In order to respond well to specific applications, Chi and BC or KBC have often had their properties modified by impregnating, casting, blending, or mixing together, or with different biopolymers, as well as directly adding these reinforcements to the culture media of cellulose synthesis bacteria [23,41,42]. Among those common derivatives, the interesting structures, multifunctional properties, eco-friendliness, and biocompatibility of Chi/BC or KBC blends are the main driving forces that promote the development of these green biocomposites as edible food films or coatings [5,6,11,12], pollution removal or treatment agents [7,8], wound dressing materials [1,9], drug delivery products, cell or enzyme immobilization matrices, or scaffolds in tissue engineering [3], some of which are shown in Table 1.

Nevertheless, the rheological properties of such blends have not been evaluated in-depth [53,54], despite the fact that they have strong potential to undergo procedures related to dispersing, mixing, stirring, extrusion, coating, spinning, injection molding, gelation, phase transitions, coagulation, and sedimentation, and even aging. Essentially, the rheological properties of the solutions can affect the spreadability, thickness, and uniformity of the forming film. It should be noted that both the product properties and economic effectiveness of the production are strongly dependent upon the rheology of the initial film-forming solutions [55,56], and it is anticipated that rheology will be an indispensable field for applied research for almost all elastic polymers.

In the preparation of Chi/BC or KBC blends, the dissolution acid concentration or types strongly affected the structural organization and rheological characteristics of the initial Chi solution, resulting in the differences in the structure, mechanical properties, thermal stability, water vapor permeability, and anti-microbial activity of the prepared biofilms [13,57,58]. Acetic acid at a concentration of 0.1 M or 1% is commonly used as organic acid for solubilizing Chi, with pH values slightly higher than Chi solution prepared with lactic acid. The inter- and intramolecular hydrogen bonds, or semi-crystalline structure, render Chi difficult to dissolve in water, alkali, alcohol, acetone, and most organic solvents [59]. Conversely, in acidic solutions such as acetic, hydrochloric, formic, butyric, malic, citric, lactic, oxalic, propionic, and succinic acids, amine groups on the Chi molecule chains are protonated to ${\mathrm{NH}}_{3}^{+}$, resulting in the destruction of the hydrogen-bonded networks by electrostatic repulsion between positive charges, leading to the dissolution process of Chi [13,58,60]. Furthermore, the rheological characteristics of Chi/BC or KBC variants are strongly affected by temperature, hydrostatic pressure, and the amount of BC or KBC present. Therefore, the present study focuses on investigating the rheological properties of Chi solutions modified by KBC in order to specify the affect of preparation condition and concentration of KBC on the viscosity of the prepared solutions, as well as the characteristics of these formed Chi/KBC biopolymer films.

## 2. Materials and Methods

### 2.1. Materials

Chitosan, from squid (Chi, molecular weight 580,000 g·mol^−1^ and degree of deacetylation 90%) was purchased from Pol-Aura (Dywity, Poland). Sodium hydroxide (NaOH), acetic acid (CH_3_COOH), and lactic acid (C_3_H_6_O_3_) were purchased from Chempur (Piekary Śląskie, Poland). Sucrose was supplied by Amersco LLC (Framingham, MA, USA). All reagents were used without further purification. Waste whey was collected from Kromilk A.S (Kromeriz, Czech Republic). Black tea was purchased from a grocery store in Zlin, Czech Republic.

The KBC used was synthesized at the laboratory of the Centre of Polymer Systems, University Institute, Tomas Bata University in Zlin, Czech Republic via kombucha fermentation of waste whey, black tea, and sucrose using *Komagatacibacter xylinus* CCM 3611 for 15 days under static culture conditions at 30 °C, as shown in our previous studies [38,61]. Harvested KBC membranes were treated by immersion in 0.5% *w*/*v* NaOH at 80 °C for 1 h (volume of biopolymer mass and volume of alkaline solution were taken in a 1:2 ratio), accompanied by triplicate washing with double distilled water, over-drying at 40 °C until a constant weight was reached, and milling for 1 min at room temperature to fine granular powder using a micro ball mill (Lab Wizz 320, Laarmann Group, Roermond, The Netherlands) under a frequency rate of 25 Hz. The concentration of KBC was then determined based on its dry weight percentage as the main ingredient of the solutions and films prepared.

### 2.2. Preparation of Chi/KBC Solutions and Films

Chi (2% *w*/*v*) was completely dissolved in acetic or lactic acid (0.1 M) for 24 h at room temperature. KBC powder was then added with the ratios of 0, 1, 2, 5, and 10% *w*/*w*. Each 40 mL of Chi/KBC film-forming solution was stirred at 100 rpm for 24 h before being studied for rheological behavior.

To achieve biofilms, 40 mL Chi/KBC film-forming solutions were poured into squared Petri dishes (100 × 15 (mm)), air-dried at room temperature for 3 days with Chi dissolved in acetic acid (Chi (acetic acid)/KBC), and for 7 days with Chi dissolved in lactic acid (Chi (lactic acid)/KBC). Subsequently, the samples were stored in a dehumidifier and used for further analysis.

### 2.3. Rheological Study

The rheological behavior of Chi/KBC solutions was measured at 25, 30, 35, and 40 °C using a Bohlin Visco 88 viscometer (Marlvern, Panalytical, Malvern, UK). The measurement cell consisted of a concentric cylinder with an inner diameter of 17 mm. Rheological curves were obtained after 5 min stabilization, and the heating rate was 0.2 °C/min. The shear stress (τ) was determined as a function of shear rate (γ) from 18 to 1230 s^−1^, and a minimum range of 1.5%. The testing solution temperature remained constant during the measurement via the use of a water bath circulator directly installed on the device. Experimental data were fitted to the Arrhenius equation (Equation (1)) and the Ostwald de Waele model (power-law) (Equation (2)) to determine the values of the activation energy of flow (*E_a_*), preexponential factor (*A*), the consistency index (*k*), and the flow behavior index (n).

(1) $$\eta =Aexp\left(\frac{E_{a}}{RT}\right)$$

where *η* is the viscosity of the Chi solution modified by KBC (Pas), *A* is a preexponential factor (1/s), *E_a_* is the activation energy of flow (kJ/mol), *R* is the gas constant (kJ/mol K), and *T* is the absolute temperature (K).

τ = kγ^n^ (2)

where τ is shear stress (Pas), γ is shear rate (1/s), n is a rheological parameter known as a non-Newtonian index (dimensionless), and k corresponds to consistency index (Pa·s^n^).

### 2.4. Atomic Force Microscope (AFM)

AFM was used to explore the surface of the prepared Chi/KBC biofilms. The scans were performed on a scanning probe microscope (Veeco, Digital Instrument, Santa Barbara, CA, Santa Barbara, CA, USA), with a resonant frequency of 110 kHz and a nominal tip radius of 10 nm. All recordings were conducted under standard room conditions (RH 30%, temperature 25 °C), with a scan rate of 0.5 Hz and resolution of 512 × 512 points, without additional data filtration.

### 2.5. Fourier-Transformed Infrared Spectroscopy (FTIR)

FTIR analysis was performed to examine the chemical groups and structures of the Chi/KBC biopolymer films using a Nicolet iS10 spectrometer (Thermo Scientific, Waltham, MA, USA) fitted with an attenuated total reflectance mode (iD5-Ge-ATR) assembly. The samples were scanned at a 4.0 cm^−1^ resolution using 64 scans in the wavenumber range of 400–4000 cm^−1^.

### 2.6. Thermogravimetric Analysis

The thermal stability capacity of the Chi/KBC films was analyzed using a TGA Q500 (TA Instruments, New Castle, PA, USA). The sample weigh was in the range of 6.5–7.0 mg. The analyzed temperature was increased from 25 to 600 °C under a nitrogen atmosphere at a heating/cooling rate of 10 °C/min and a flow rate of 40 to 60 mL/min.

### 2.7. Mechanical Analysis

In order to investigate the mechanical properties of Chi/KBC films, the elastic modulus (GPa) and elongation at break (%) were determined as a function of displacement by applied force using Zwick Roell (Ulm, Germany) under a static load of 10 kg and a crosshead speed of 50 mm/min at room temperature (25 °C). The testing was conducted following the ASTM standard method D882 [62].

### 2.8. Statistical Analysis

All measurements were recorded in triplicate, and the results were reported as mean ± standard deviation. Analysis of variance (ANOVA) was applied for statistical evaluation, and experimental results were displayed as mean ± standard error, where *p* < 0.05 was determined as statistically significant.

## 3. Results

### 3.1. Rheological Data and Viscosity of Chi/KBC Film-Forming Solutions

**Figure 1:**
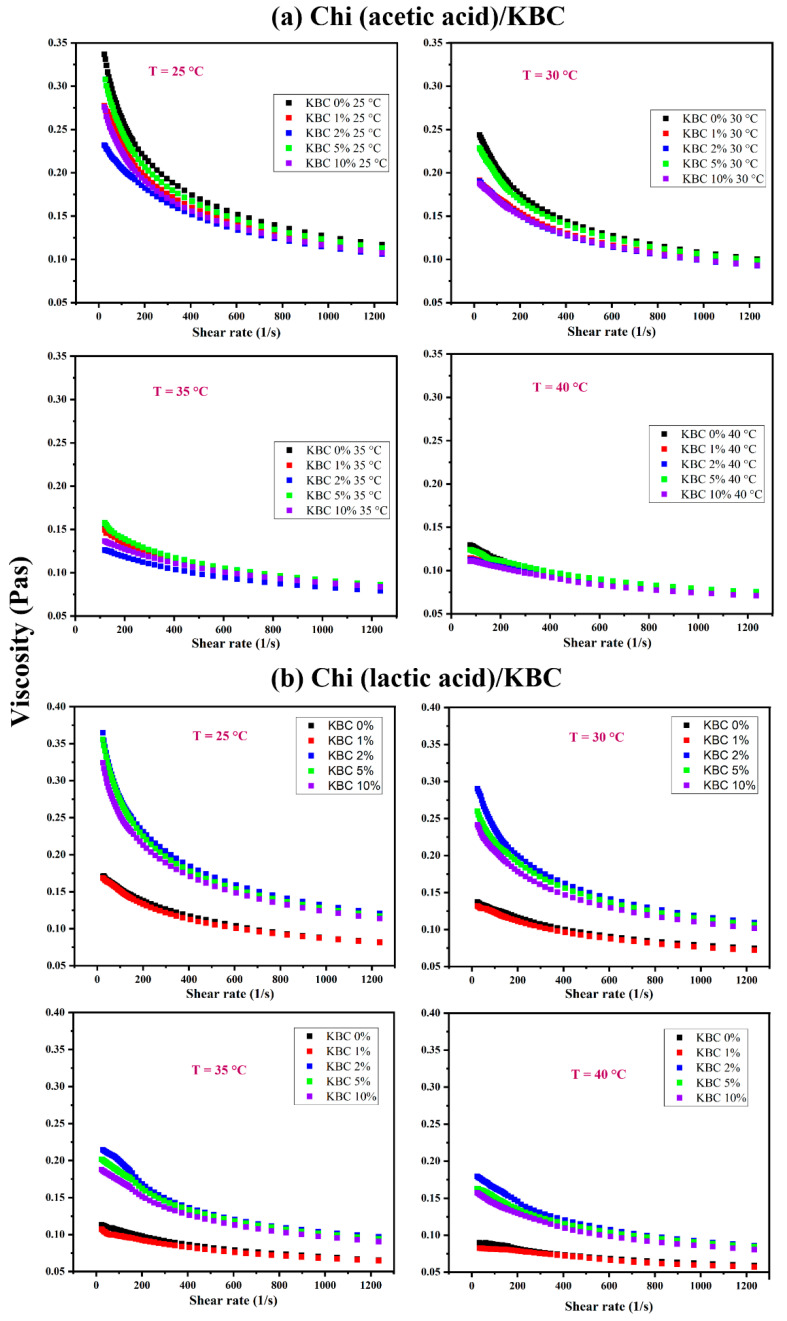
Variation of the viscosity of Chi dissolved in (**a**) acetic acid and (**b**) lactic acid containing various KBC concentrations at 25, 30, 35, and 40 °C.

Figure 1 shows a significant difference in the rheological behavior of Chi/KBC film-forming solutions in differently dissolved solvents, different temperatures, and various KBC concentrations. In differently dissolved acid types, interaction patterns between chitosan and acids mainly include electrostatic interactions, hydrogen bonds, and hydrophobic interactions. In Chi (acetic acid)/KBC samples, Chi is partially crystalline and consist mainly of ionic interactions. In contrast, in Chi (lactic acid)/KBC samples, Chi is amorphous, with the existence of both ionic interactions and hydrogen bonding [58,60,63]. These factors substantially explain the higher viscosity values, as well as the lower viscosity reduction percentage when comparing Chi (lactic acid)/KBC samples and Chi (lactic acid)/KBC film-forming solutions (Table 2).

Regarding temperature issues, the viscosity of all examined solutions decreased when the temperature increased. It is possible for the viscosity reduction percentages to be over 50 and 60% as the temperature rises from 25 °C to 40 °C for both Chi (acetic acid)/KBC and Chi (lactic acid)/KBC, as shown in Table 2. Essentially, solution viscosity is formed by the adhesive forces between molecules. With thermal increase, the thermal energy of the molecules in the testing solutions increased, even overcoming the adhesive forces, which led to them moving more freely and the intermolecular distances being extended (called the thermal expansion associated with Brownian motion). Ultimately, their collision frequency was greater, their resistance against the flow was higher, and their total intermolecular linking forces were lower, leading to a decrease in the investigated solutions’ viscosities. For the effect of KBC concentrations, considered at each temperature (25, 30, 35, and 40 °C), the results exhibited a decrease in the viscosity of all analysis solutions due to the presence of KBC powder with the sequential reduction ratio in the samples from non-KBC ≥ 10 > 1 ≥ 2 ≥ 5 (% *w*/*w*) of Chi (acetic acid)/KBC samples. This result was completely in contrast to Chi (lactic acid)/KBC samples, with a viscosity increasing from non-KBC ≤ 1 < 2 ≤ 5 ≤ 10 (% *w*/*w*) samples. In the case of Chi (acetic acid)/KBC, the viscosity values were decreased, which was ascribed to KBC presence, causing several collisions and cleavages of intramolecular bonds to shorten the linear chain of the glucosamine groups of the chitosan matrix, leading to a decrease in the pure viscosity of these modified composite solutions. Conversely, in the increase of the viscosity obtained in Chi (lactic acid)/KBC samples, these displayed results were often related to the rise in solid particle concentration (KBC powder) in the solution followed by a limit in the movement, collision, or cleavage. Another point worth noting is that KBC possesses an OH-rich nature and high hydrophilicity. The increases in the viscosity of the prepared solution were also attributed to high sufficient KBC concentrations having increased hydrogen bonding between the hydroxyl groups of KBC and the amine and carboxylic groups of Chi. Herein, the sample containing 1 (% *w*/*w*) KBC showed an insignificant difference in viscosity compared to the control sample (Figure 1b). This might be due to the lower presence of KBC, which is unable to cause dramatic effects on the viscosity of this measured solution.

The interaction between temperature and the viscosity of the prepared solutions was illustrated via the Arrhenius equation, and the values of constants *E_a_* and A have been calculated, as shown in Table 3. As observed, there was a difference in the magnitudes of activation energy, *E_a_*, at different KBC concentrations, which were similar to the viscosity measured results exhibited in Figure 1. This has confirmed the results used to calculate the viscosity of modified Chi/KBC solutions at a specific measured temperature. Expressly, taking into consideration the Chi (acetic acid)/KBC samples, the activation energy, *E_a_*, of non-KBC and 5 (% *w*/*w*) KBC samples were higher than the remaining samples. This phenomenon was attributed to their higher viscosity (Figure 1a), which required more space or a more extensive track for the molecules in these solutions to flow into; therefore, their activation energy, *E_a_*, was higher. Similarly, for Chi (lactic acid)/KBC samples, the viscosity of non-KBC and 1 (% *w*/*w*) KBC samples were low (Figure 1b); consequently, their activation energy was also lower.

For the evaluation of another aspect, it is evident from Figure 1 that the viscosities of the testing samples have significantly decreased with increasing shear rates. This relationship revealed that the investigated solutions exhibited non-Newtonian behavior. As such, to model the viscosity performances of these solutions under the concurrent influence of temperature and KBC concentration (both the temperature and concentration will change during the process), the Ostwald de Waele model (power-law) was applied, and the computation results of the constants of the model are presented in Table 4. The value of constants was as follows: “n” was close to 1, and “k” fluctuated over a wide range (0.73–0.99 and 0.14–0.89 for Chi (acetic acid)/KBC, or 0.71–0.96 and 0.12–0.99 for Chi (lactic acid)/KBC). This revealed that the prepared solution displayed shear thinning behavior. These results indicate that the temperature and KBC concentration have a strong effect on the viscosity of Chi solutions modified by KBC. Generally, this model will be useful for the manufacturing procedure of biopolymer films, scaffolds, bio-printing inks, and products related to injection or direct extrusion through a needle, using Chi solutions dissolved in acetic or lactic acid and modified by KBC powder. Nevertheless, it should be noted that the obtained constants in Table 4 are only suitable for the range of evaluated temperatures and concentrations.

### 3.2. Characterization of Chi/KBC Biopolymer Films

#### 3.2.1. Morphology Analysis

**Figure 2:**
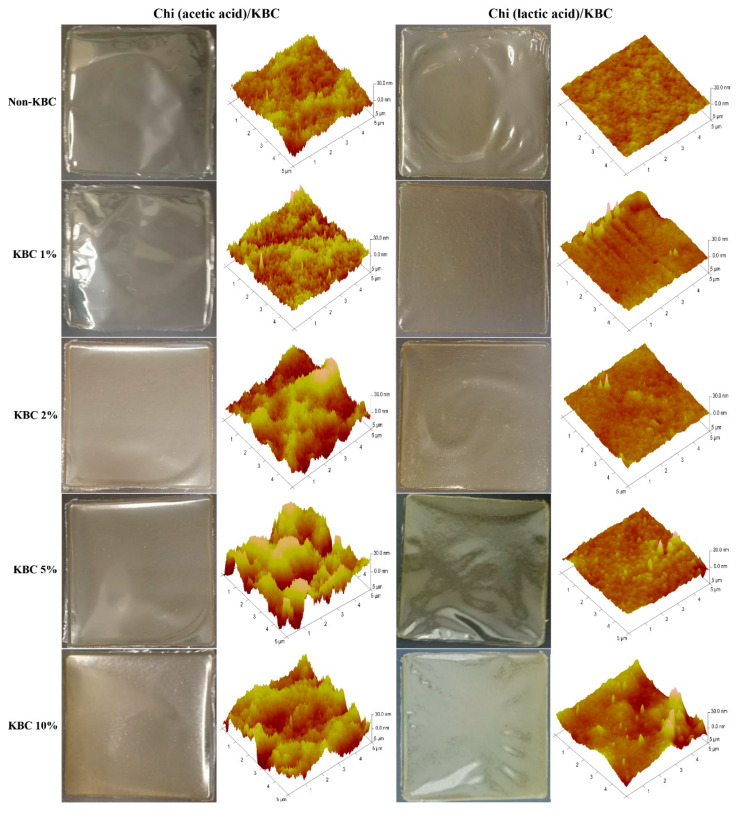
Physical appearance and AFM topography of modified Chi films at various concentrations of KBC.

The physical appearance and topography of the prepared Chi/KBC films can be seen in Figure 2. In brief, these biopolymer films were transparent, explicit, opalescent, or pale yellow, with considerably flexibility. The results from AFM suggested that the surface characteristics of the Chi (lactic acid)/KBC films were smoother than the Chi (acetic acid)/KBC samples, clearly displayed by the samples of non-KBC. The interaction capacity between Chi and dissolved acid was the main reason for these dissimilarities. The pKa of lactic acid (~3.86) is lower than that of acetic acid (~4.8), resulting in the ionic interactions between the amine and carboxylic groups of Chi molecules being stronger in lactic acid compared to the same interactions in acetic acid [13,63]. Additionally, the increase of KBC presence was directly proportional to the scabrous level of the formed film topography. This phenomenon was attributed to the drying, condensation, and displacement of granulated KBC, leading to the formation of aggregates or their in-situ association on Chi substrates.

#### 3.2.2. Chemical Structure Analysis

**Figure 3:**
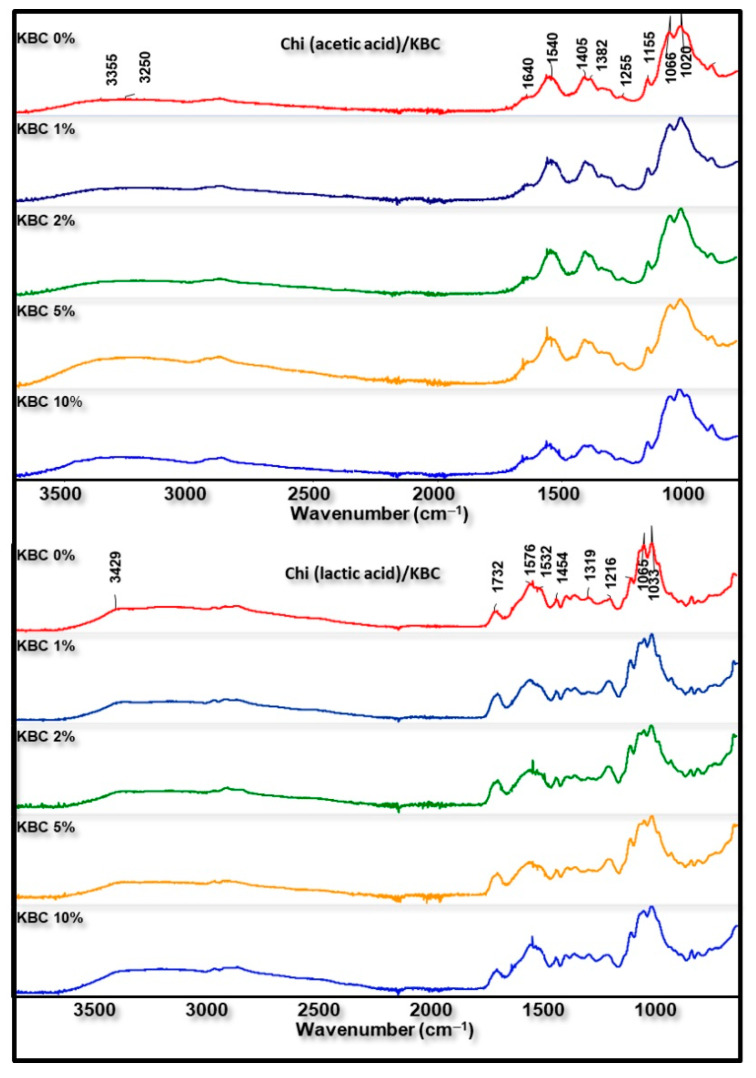
FTIR spectra of prepared films (Chi (acetic and lactic acid)/KBC) at various concentrations of KBC.

FTIR analysis was used to examine the structural differences of Chi/KBC films. All of the characteristic peaks of neat Chi, KBC, acetic, and lactic acid were observed in the prepared related biopolymer films (Figure 3): essentially, the bands near 3328 cm^−1^ (N-H and O-H stretching), 2925 cm^−1^ (C-H asymmetric stretching in methyl, methylene, or methoxy groups), and 1640 cm^−1^ (C=O bending and N-H stretching) from Chi, KBC, acetic, or lactic acid. Parallel to that, the vibrations at 1394–1332 cm^−1^ are ascribed to amine groups (-NH bending); those at 1020–1195 cm^−1^ relate to C-O-C symmetric stretching; and C-N, C-C, and C-O stretching form used raw materials. The bands at 1125, 1216, and 1732 cm^−1^ are characteristic bands of lactic acid, which reveal the presence of lactic acid in all Chi blend films.

Nevertheless, slight differences were still recorded in the FTIR spectra of these analyzed biopolymer films. As can be seen from Figure 3, a higher density of the peaks at the region around 3300–3400 cm^−1^ was detected in the analysis result of biomaterial containing KBC compared to pristine materials. This phenomenon is ascribed to the extra-presence of KBC with varying content in the composition of finished biopolymer films. Furthermore, the insignificant difference between the spectra of chitosan modified by KBC and neat materials has also been observed. These variations might be due to some transformations in the original properties under the combination and dissolution process of the ingredients together. In addition, the peaks in the area 1400–1700 cm^−1^ slightly deviated, with a higher wavenumber observed in Chi (lactic acid)/KBC samples compared to Chi (acetic acid)/KBC samples, which is attributed to the amide II of chitosan shifted to a higher frequency when the lactic acid was used to dissolve Chi instead of acetic acid. These results were in close agreement with the results obtained by Phatchayawat et al., 2022; Liu et al., 2022; Qiao et al., 2019; and Lin et al., 2013 [3,4,8,13].

#### 3.2.3. Thermogravimetric Analysis

**Figure 4:**
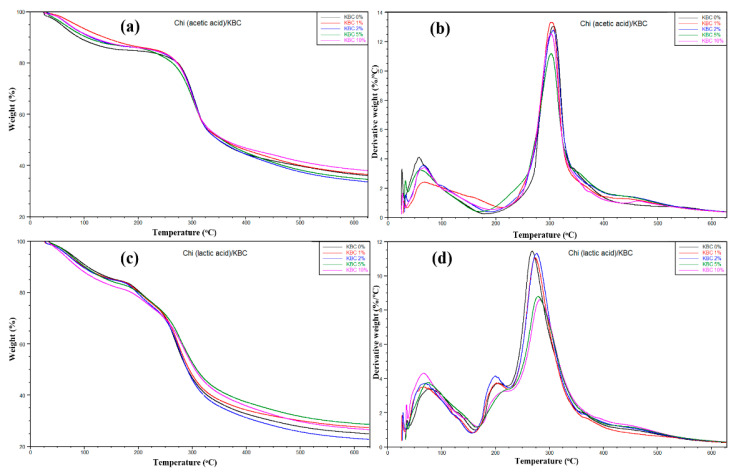
Plots of (**a**,**c**) TGA and (**b**,**d**) DTG of prepared films (Chi (acetic and lactic acid)/KBC) at various concentrations of KBC.

The thermal behaviors of Chi/KBC films are shown in Figure 4, with insignificant different stability in thermal degradation behavior via the TGA and DTG curves. In the beginning, a slight weight loss in the prepared samples was observed when the temperature grew from 25 to 180 °C. This loss was attributed to the water releasing process of the prepared biofilms by the hydrophilicity natural KBC and ${\mathrm{NH}}_{3}^{+}$ groups or the degree of protonation of Chi ingredients. Herein, this similarity also indicates that the moisture content of the samples is the same. Subsequently, a sharp weight loss has appeared in the increasing temperature from 180 to 360 °C, related to the decomposition and deacetylation or evaporation of residual acids of Chi and KBC composition. Finally, forming carbon char took place when the temperature was over 360 °C. These results closely correspond to the DTG curves of the samples (Figure 4b). It is easily seen that there are two prominent peaks for both Chi (acetic acid)/KBC and Chi (lactic acid)/KBC films. One peak is around 80 °C and exhibited water evaporation, and the other peaks revealed polymer decomposition, around 200 and 300 °C, respectively.

#### 3.2.4. Mechanical Analysis

**Figure 5:**
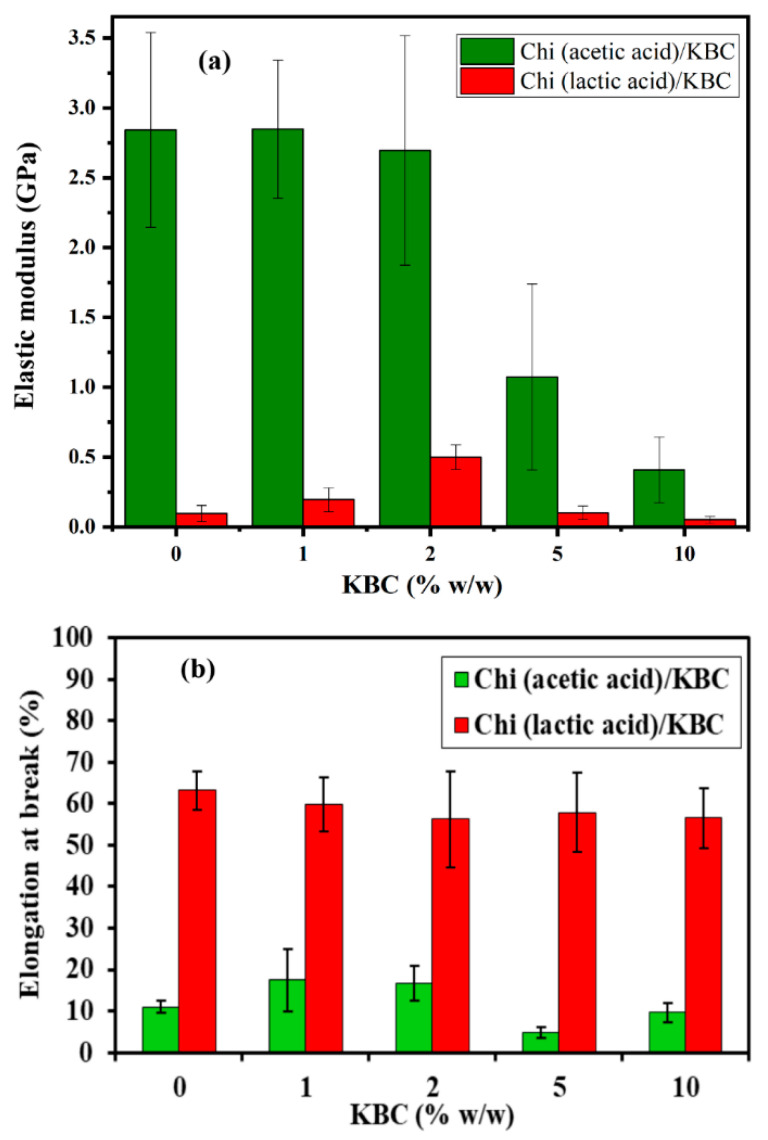
(**a**) Elastic modulus and (**b**) Elongation at break of Chi films modified by KBC; bars represent mean ± SD (standard deviation).

The mechanical strength of the Chi/KBC films was examined via the values of elastic modulus and elongation at break and are presented in Figure 5. Chi (lactic acid)/KBC films exhibited superior resilience at all evaluated concentrations, particularly the values of elastic modulus and elongation of break in the range 0.052–0.196 (GPa) and 56.8–63.2 (%) compared to 0.409–2.848 (GPa) and 9.6–17.5 (%) of Chi (acetic acid)/KBC. This phenomenon was attributed to the interactions between Chi and the lactic acid, which was stronger than the acetic acid, leading to higher elasticity and percentage of elongation at break with Chi (lactic acid)/KBC samples. In addition, the molecular weight of Chi dissolved in acetic acid was more extensive than that dissolved in lactic acid, resulting in Chi (acetic acid)/KBC films often presenting a pattern similar to brittle materials that are different from Chi (lactic acid)/KBC films, which have a rubber-like quality [13,63,64]. In brief, with the selection of a proper solvent for Chi solubilization, it may be possible to improve the performances of Chi/KBC variants for certain special applications.

Regarding the influence of the granulated KBC ingredient on the mechanical properties of Chi/KBC films, the measurements revealed that the most positive results were recorded at KBC 10% *w*/*w*, with the elasticity higher than the control samples (6.94-fold and 1.85-fold better for biopolymer films dissolved in acetic and lactic acid, respectively). In addition, it should be emphasized that this enhancement effectiveness was insignificant in the majority of cases of KBC in low concentrations (1 and 2% *w*/*w*), even causing a reverse effect with the samples of Chi (lactic acid)/KBC, from which it might be implied that KBC has a strong contribution to the durability of the prepared films [23].

## 4. Conclusions

The Arrhenius equation and the Ostwald de Waele model (power-law) were applied to model the rheological behaviors of Chi/KBC film-forming solutions. Activation energy of flow (*E_a_*), preexponential factor (A), non-Newtonian index (n), and consistency index (k) were calculated, and the results revealed that these investigated solutions exhibited a non-Newtonian behavior. Temperature and KBC concentration all strongly affected their viscosity, and with the selection of a proper solvent for chitosan solubilization, it may be possible to improve the performances of chitosan films for certain special applications. These obtained models can be useful in the preparation process of the solutions of Chi dissolved in acetic or lactic acid modified by KBC powder. In addition, FTIR, TGA, and mechanical analysis results of the biopolymer films have confirmed the presence of Chi, bacterial cellulose, acetic, and lactic acid as the corresponding components in the products possessing the elasticity excessive control samples, especially in the samples containing KBC 10% *w*/*w*. This research also promoted some further tests on the influence of the molecular weight of Chi, polydispersity, and particle size of KBC powder or other cellulose sources (such as leaves, wild plants, rice husks, banana fibers, and coconut fibers), to move towards the complete rheological models of Chi-based green biocomposites.

## Figures and Tables

**Table 1:** List of recent research regarding the combination of Ch with BC or KBC and desired application.

Materials	Preparation Method	Enhanced Properties	Desired Application	Ref.
*Chi*/*KBC*	Impregnation	Water vapor permeability; antioxidant activity; against ultraviolet	Active food packaging	[5]
*Chi*/*BC*	Casting	Mechanical properties	Food packaging	[6]
*Chi*/*BC*/metal-organic framework	Impregnation	Water stability	Wastewater treatment	[7]
*Chi*/*BC*/magnetic attapulgite	Blending	Adsorption capability	Water treatment	[8]
*Chi*/*BC*	Grafting	Uniform; acid and temperature stability	Papermaking; Food packaging; Textiles; Medical	[43]
*Chi*/*BC*	Blending	Thermal stability	Immobilize proteins	[44]
*Chi*/*BC*/glycerol /carboxymethyl cellulose	Casting	Water vapor transmission rate; tensile strength	Antimicrobial films	[45]
*Chi*/*BC*/ ciprofloxacin	Impregnation	Antimicrobial activity	Wound dressing	[46]
*Chi*/*BC*/silver sulfadiazine	Impregnation	Mechanical and antibacterial properties	Food packaging, Tissue engineering, Drug delivery, Biomedical	[47]
*Chi*/*BC*	Impregnation	Antimicrobial activity; porosity; migration of cell	Chronic wound healing agents	[48]
*Chi*/*BC*/ZnO	Blending	antimicrobial activity; thermal stability; Compressive strength	Antibacterial dressing	[49]
*Chi*/*BC*/poly(N-isopropylacrylamide/polyvinyl alcohol/methyl oleate/silver sulfadiazine	Blending	Mechanical strength and biocompatibility	Wound dressing materials	[50]
*Chi*/*BC*/Poly(vinyl alcohol)	Casting	Tensile strength and antibacterial properties	Food packaging	[51]
*Chi*/*BC*/collagen	Impregnation	Breathability and antibacterial properties	Wound dressing	[52]

**Table 2:** The viscosity reduction percentage of Chi/KBC film-forming solutions as temperature increases from 25 °C to 40 °C.

KBC (% *w*/*w*)	Viscosity Reduction Percentage (%)	
	Chi (Acetic Acid)/KBC	Chi (Lactic Acid)/KBC
0	60.53	51.38
1	58.22	51.54
2	57.84	53.43
5	57.81	54.21
10	59.42	54.63

**Table 3:** The parameters of Arrhenius equation of Chi solutions modified by KBC with different concentrations.

KBC (% *w*/*w*)	Chi (Acetic Acid)/KBC			Chi (Lactic Acid)/KBC		
	*E_a_* (kJ/mol)	A (1/s)	*r^2^*	*E_a_* (kJ/mol)	A (1/s)	*r^2^*
0	13.67	0.054 × 10^−6^	0.9989	10.80	2.46 × 10^−6^	0.9994
1	11.95	0.25 × 10^−6^	0.9955	10.26	1.55 × 10^−6^	0.9996
2	11.43	0.28 × 10^−6^	0.9874	11.75	1.23 × 10^−6^	0.9977
5	12.13	0.22 × 10^−6^	0.9999	11.72	4.43 × 10^−6^	0.9962
10	11.79	0.28 × 10^−6^	0.9972	11.52	4.99 × 10^−6^	0.9997

**Table 4:** The combined effect of temperature and KBC concentration on the viscosity of modified Chi solutions.

Chi (Acetic Acid)/KBC												
KBC (% *w*/*w*)	25 °C			30 °C			35 °C			40 °C		
	k	n	*r^2^*	k	n	*r^2^*	k	n	*r^2^*	k	n	*r^2^*
0	0.89	0.73	0.9952	0.57	0.77	0.9946	0.57	0.77	0.9946	0.24	0.85	0.9952
1	0.69	0.76	0.9944	0.29	0.88	0.997	0.14	0.99	0.9963	0.18	0.90	0.9954
2	0.59	0.80	0.9939	0.38	0.81	0.9952	0.23	0.87	0.9964	0.18	0.89	0.9954
5	0.81	0.74	0.9958	0.52	0.78	0.995	0.35	0.82	0.9923	0.25	0.85	0.9954
10	0.67	0.76	0.9961	0.37	0.82	0.9951	0.26	0.86	0.9953	0.18	0.88	0.9962
**Chi** **(Lactic Acid)/KBC**												
0	0.35	0.81	0.9944	0.26	0.84	0.996	0.26	0.84	0.996	0.15	0.88	0.9977
1	0.35	0.81	0.9952	0.200	0.89	0.9981	0.12	0.96	0.999	0.12	0.91	0.9963
2	0.98	0.72	0.9953	0.73	0.75	0.9946	0.51	0.78	0.9908	0.35	0.82	0.9917
5	0.99	0.71	0.9944	0.59	0.78	0.994	0.43	0.80	0.9924	0.32	0.83	0.9948
10	0.84	0.73	0.9957	0.55	0.78	0.9944	0.38	0.82	0.9923	0.29	0.84	0.9942

## Data Availability

Data are contained within the article.
